# Revised role for Hfq bacterial regulator on DNA topology

**DOI:** 10.1038/s41598-018-35060-9

**Published:** 2018-11-14

**Authors:** Antoine Malabirade, David Partouche, Omar El Hamoui, Florian Turbant, Frédéric Geinguenaud, Pierre Recouvreux, Thomas Bizien, Florent Busi, Frank Wien, Véronique Arluison

**Affiliations:** 1 Laboratoire Léon Brillouin LLB, CEA, CNRS UMR12, Université Paris Saclay, CEA Saclay, 91191 Gif-sur-Yvette, France; 2 Synchrotron SOLEIL, L’Orme des Merisiers, Saint Aubin BP48, 91192 Gif-sur-Yvette, France; 3 UFR SMBH, Université Paris 13, Sorbonne Paris Cité, 93017 Bobigny, France; 4 Aix Marseille Université, CNRS, IBDM, Marseille, France; 5 Unité de Biologie Fonctionnelle et Adaptative, CNRS UMR8251, Université Paris Diderot, 75013 Paris, France; 6 Université Paris Diderot, Sorbonne Paris Cité, 75013 Paris, France

## Abstract

Hfq is a pleiotropic regulator that mediates several aspects of bacterial RNA metabolism. The protein notably regulates translation efficiency and RNA decay in Gram-negative bacteria, usually *via* its interaction with small regulatory RNA. Besides these RNA-related functions, Hfq has also been described as one of the nucleoid associated proteins shaping the bacterial chromosome. Therefore, Hfq appears as a versatile nucleic acid-binding protein, which functions are probably even more numerous than those initially suggested. For instance, *E. coli* Hfq, and more precisely its C-terminal region (CTR), has been shown to induce DNA compaction into a condensed form. In this paper, we establish that DNA induces Hfq-CTR amyloidogenesis, resulting in a change of DNA local conformation. Furthermore, we clarify the effect of Hfq on DNA topology. Our results evidence that, even if the protein has a strong propensity to compact DNA thanks to its amyloid region, it does not affect overall DNA topology. We confirm however that *hfq* gene disruption influences plasmid supercoiling *in vivo*, indicating that the effect on DNA topology in former reports was indirect. Most likely, this effect is related to small regulatory sRNA-Hfq-based regulation of another protein that influences DNA supercoiling, possibly a nucleoid associated protein such as H-NS or Dps. Finally, we hypothesise that this indirect effect on DNA topology explains, at least partially, the previously reported effect of Hfq on plasmid replication efficiency.

## Introduction

The hexameric Hfq is an abundant and phylogenetically conserved protein present in about half of bacteria and in some archaeal species. Consistent with its high cellular level, the protein coordinates multiple roles inside the bacterial cell. For instance, the initial description of Hfq was associated with its involvement in the activity of bacteriophage Qβ replicase (hence its name Host factor for bacteriophage Qβ^1^). The pleiotropic functions of Hfq were however highlighted later when the *hfq* gene was disrupted in *Escherichia coli*, decreasing bacterial growth rate, changing mutagenesis rate, increase in UV sensitivity, oxidant and osmo-sentivity, as well as a decreasing plasmid supercoiling^2,3^. Most of these *hfq*-null phenotypes are due to Hfq-dependant small noncoding RNA (sRNA) based regulations^4^. Indeed, Hfq is required to mediate sRNA stress-response^5^. This regulatory mechanism is based on the hybridisation of the sRNA to its target mRNA, therefore altering the mRNA translation and stability^6^. This effect can be either negative or positive. For instance the same sRNA can positively regulate a mRNA target, while it regulates negatively another^7–9^.

Besides, Hfq also binds DNA and has been described as one of the *E. coli* nucleoid associated proteins (NAP)^10–13^. This was notably revealed *in vivo* by cellular localisation experiments demonstrating that about 20% of Hfq was complexed with DNA within the bacterial nucleoid^13–15^.

Interestingly, Hfq is structurally related to the Sm eukaryotic family of proteins, which participates in nucleic acid-related processes, including splicing, telomeres replication, RNA decapping and decay^16,17^. Indeed, the amino-terminal region of Hfq (about 65 amino acid residues) folds similarly to Sm proteins. This region comprises a bent antiparallel β-sheet, capped by an N-terminal α-helix. The β-sheets from six monomers interact with each other to assemble in a toroidal structure^18,19^. Although the mechanism by which Hfq binds nucleic acids is not fully understood, it is now well-established that the inner pore on the proximal face of the torus (on which the α-helix is exposed) binds U-rich RNA, that A-rich sequences bind to the distal face, and that the lateral rim also binds A/U-rich sequences^20–22^. The distal face and the rim of the protein are involved in both DNA and RNA binding, while the proximal face seems to be involved in RNA fixation only^11,20^. Besides its Sm-like domain, Hfq C-terminal region (CTR) also plays a role in nucleic acid recognition^23,24^. We previously showed that Hfq and precisely its CTR bridges distant regions of plasmids^12,13^ and highlighted that it induces a strong compaction of DNA^24^. Note that DNA compaction results from a synergy of different mechanisms, including DNA-bridging, DNA-bending, DNA-supercoiling, self-assembly of nucleoid proteins, as well as phase separation^25,26^. Thus Hfq DNA-bridging may just be one contributor to nucleoid compaction. 3D-structures of various Hfqs have been resolved^21,22,27–30^, all lack the CTR and the way this region folds remains unknown so far. It has however been demonstrated that Hfq is able to self-assemble and that this characteristic is dependent on the presence of CTR, an observation in accordance with the observation that the flexible CTR could facilitate protein:protein interactions^31,32^. Indeed, it has recently been shown that the CTR region of *E. coli* Hfq forms an amyloid-like structure^33–35^, explaining why the protein is able to self-assemble *in vitro* and *in vivo*^12,31,33,36^. While the same region of Hfq is responsible of its propensity for self-assembly, DNA bridging and compaction^12,24^, the relation between these properties remains unclear. Both the architectural bridging properties of nucleoid associated proteins (NAPs) and their ability to self-associate may have a role on bacterial DNA condensation^25^.

So far, only a few studies have shed light on the role of Hfq in DNA metabolism^13^, *e.g*. it has been shown to influence plasmid negative supercoiling *in vivo*^2^, replication efficiency^37^ and some studies have shown a role in transcriptional activity^38–40^. Some of the phenotypic effects due to the lack of Hfq may be attributed to defects in DNA-related processes; filamentation or slowed-down bacterial growth are for instance a sign of replication deregulation, sensitivity to mutagens could indicate unreliable DNA repair. Motivated by this unsolved role of Hfq in DNA-related processes, we have investigated how Hfq interaction with DNA affects the nucleic acid structure^24^. In this work, we focus our attention on two aspects of DNA:Hfq interaction, how binding to DNA induces Hfq self-assembly and how Hfq affects DNA structure and topology. Our results enable us to propose a mechanism underlying the function of Hfq in DNA packaging and its precise influence on plasmid supercoiling.

## Results

*E. coli* Hfq:DNA complex has been analysed previously *in vitro* by molecular imaging (transmission electron TEM and atomic force AFM microscopies)^12,23,24^. This allowed us to observe that Hfq tends to bridge two double-stranded distant DNA sections together, through its CTR amyloid-like region^12,23,24^.

### DNA induces amyloidogenesis of Hfq CTR

**Figure 1:**
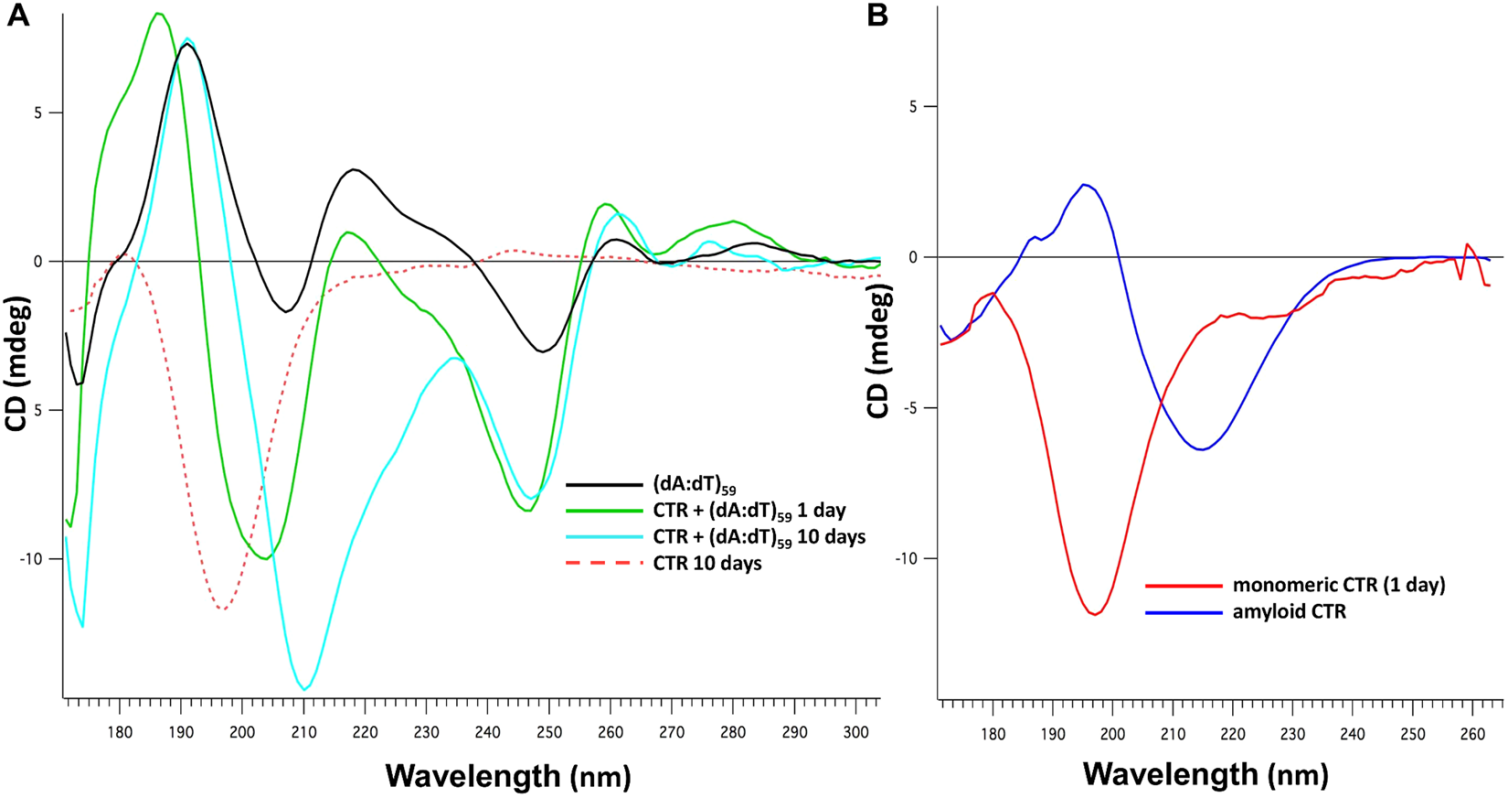
SRCD kinetics of Hfq-CTR self-assembly in the presence of DNA. SRCD experiment was performed as a function of time in order to observe progressive amyloid-like structuring. (**A**) (dA:dT)_59_: black line; CTR + (dA:dT)_59_ 1 day: green line; CTR + (dA:dT)_59_ 10 days: cyan line; monomeric CTR 10 days: dashed red line monomeric. (**B**) Controls: CTR 1 day: red line; amyloid CTR: blue line. As shown, the typical signal of amyloids at 210–220 nm appears after 7 days, while it takes more than 2 weeks with peptide alone. Note that DNA induces a structural change of the protein and conversely (see Fig. 6). For this reason we do not substract the spectrum of the peptide alone from the spectrum of DNA:peptide complex.

First, we focused our attention on the effect of DNA on Hfq self-assembly. Indeed, Hfq binding to DNA may be due to simple DNA binding of individual monomers or a cooperative self-assembly. A related question concerns the effect of DNA on amyloidogenesis of Hfq. Hfq-CTR self-assembly *in vitro* is quite a slow mechanism^33,34^, and cellular co-factors probably help to accelerate this process. Such an effect has already been demonstrated for lipids^41^. In our analysis we used Synchrotron Radiation Circular Dichroism (SRCD), which allows extending the wavelength range down to 170 nm for the identification and distinction of amyloid proteins peaks^42^. Aggregation into β-sheets in an amyloidal structure implies a significant SRCD spectral change: negative band shift from ~200 nm to ~210 nm. As shown on Fig. 1, the red-shift observed upon DNA binding proves that the amyloid structure formation is induced by the interaction with DNA, while the same peptide in the absence of DNA remains unassembled even after 2 weeks.

**Figure 2:**
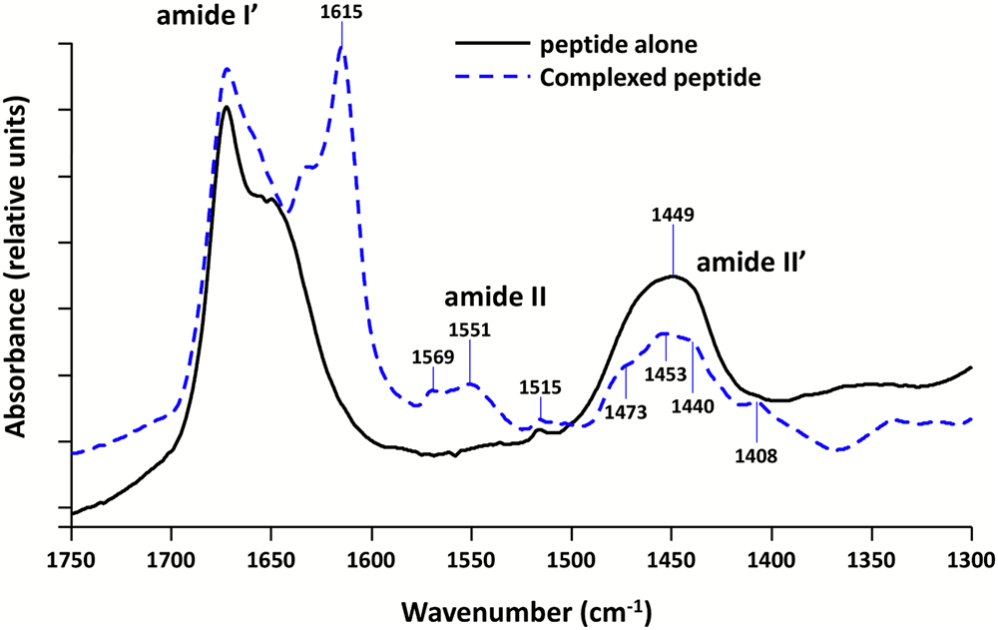
FTIR spectra of Hfq-CTR in the presence or absence of DNA. D_2_O buffered samples have been used to avoid the spectral overlaps between Amide I and water bands. Solid black line: peptide alone. Dashed blue line: difference spectrum obtained by subtracting the (dA:dT)_59_ contribution from the complex spectrum. We clearly observe in the Amide I’ band a contribution at 1615 cm^−1^, indicative of the formation of the amyloid structure in the presence of DNA. Note in the complex the presence of both Amide II and II′ bands, due to N\H and N\D in-plane bending of the peptidic groups, respectively. Strikingly, the Amide II is present only in the complex, indicating that hydrogens may be entrapped inside the complex and cannot exchange with deuterium^83^.

We confirmed this result using Fourier Transform InfraRed (FTIR) spectroscopy (Fig. 2). FTIR is useful to identify the presence of amyloid fibrils. Indeed, a peak around 1645 cm^−1^ is indicative of random coil, 1655 cm^−1^ of α-helix, and 1630–1640 cm^−1^ of β-sheet^43^. Hydrogen-bonding in cross-β structure, the fingerprint of amyloids, induces a shift to lower wavenumbers so amyloid fibrils show a β-sheet absorption band below 1620 cm^−1^ ^44^. Here D_2_O buffered samples have been used to avoid the spectral overlaps between Amide I band and strong absorption band of water at 1640 cm^−1^ (in this case Amide I/II bands are referred as Amide I’/II’). The amide I’ wavenumbers are then lowered in D_2_O environment by 5–10 cm^−1^. In Fig. 2, we clearly observe a band at 1615 cm^−1^ indicative for the formation of amyloids in the presence of DNA.

**Figure 3:**
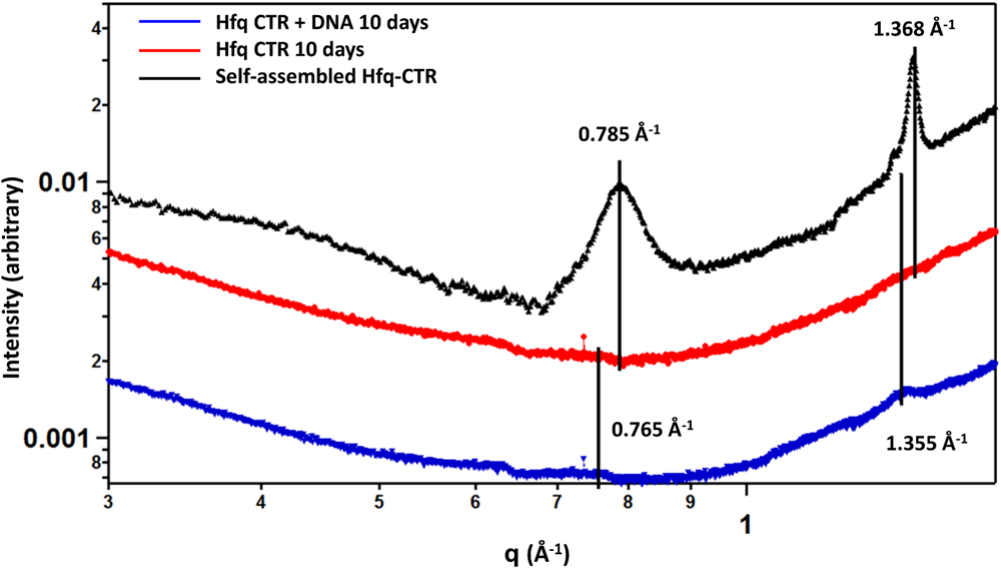
SAXS curves of Hfq-CTR in the presence or absence of DNA. β-Sheet peaks corresponding to a cross-β structure are clearly observed for Hfq-CTR in the presence of DNA (incubated 10 days, blue curve) and for Hfq-CTR alone incubated for 6 weeks at 20 mg/mL (higher time and concentration are used to ensure the formation of amyloid fibrils in the absence of cofactor, black curve). But no β-Sheet peak is observed for Hfq-CTR alone after 10 days (red curve). Peaks corresponding to cross-β structure can be seen at 0.765 and 1.355 Å^−1^ for complexed Hfq-CTR, and 0.785 and 1.368 Å^−1^ for non-complexed Hfq-CTR, corresponding to inter-sheet and inter-strand distances (d) of 8.21 and 4.63 and 8.00 Å and 4.59 Å, respectively (d(Å) = 2π/q(Å^−1^)).

Finally, the formation of the cross-β structure was also investigated by small angle X-ray scattering (SAXS) in the wide-angle region. SAXS curves are shown on Fig. 3. Weak β-sheet peaks are observed for Hfq-CTR in the presence of DNA (incubated 10 days, blue curve) and for Hfq-CTR alone incubated for 6 weeks at 20 mg/mL (to ensure the formation of amyloid fibrils in the absence of cofactor, black curve), while no β-sheet peak is observed for Hfq-CTR alone after 10 days (red curve). The peaks intensities on the blue curve are much lower than the reference (black curve) due to less incubation time resulting in less β-sheet formation. This definitely confirms that DNA promotes the formation of Hfq-CTR amyloid. Interestingly, peaks corresponding to the β-sheet structure are not the same with or without DNA. A 0.21 Å shift for inter-sheet spacing (increase from 8.00 to 8.21 Å in the presence of DNA) and a 0.04 Å shift for inter-strand spacing (increase from 4.59 to 4.63 Å in the presence of DNA) towards lower q are observed while comparing both curves. This indicates a slight change in the structure of Hfq-CTR β-Sheet arrangement upon DNA addition. Taken together, these results firmly establish that Hfq amyloid formation is induced by the interaction with DNA.

### Influence of Hfq on DNA topology

Nevertheless, the link with change in DNA structure and topology still remains unclear. It has been described previously that the linking number of plasmids isolated from cells carrying an *hfq* mutation is altered compared with DNA of WT strain^2^. This could be a direct consequence of the interaction of Hfq with DNA, or an indirect effect consecutively to a sRNA mediated regulation of other DNA-binding proteins, changing DNA topology.

**Figure 4:**
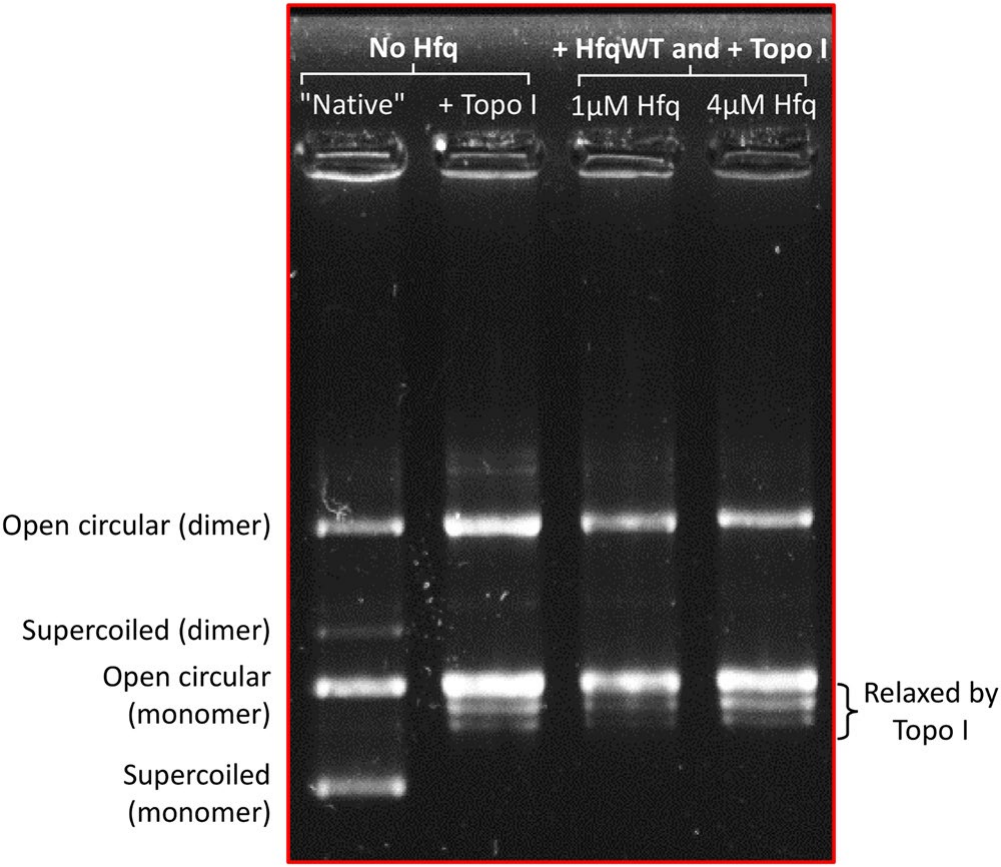
Topology gel of pHSG298 after incubation with Hfq. The presence of purified Hfq does not influence the repartition of topoisomers, confirming that Hfq has no direct effect on overall plasmid topology.

In order to distinguish between these two possibilities, we studied the interaction of purified Hfq with plasmids *in vitro*. pHSG298 plasmid was incubated with purified Hfq and then treated with calf thymus topoisomerase I to relax unconstrained supercoils. Plasmid DNA was deproteinised and electrophoresed on native agarose gels (Fig. 4). Under these conditions, the most supercoiled topoisomers migrate faster, and the relaxation induced by topoisomerase treatment slows down the migration. As shown in Fig. 4, in the absence of Hfq, plasmid DNA was completely relaxed by topoisomerase. The same result is observed when DNA is incubated with Hfq. This implies that Hfq does not constrain supercoils. This is in contrast to the effect observed for H-NS^45^. Thus, the previously reported *in vivo* effect of Hfq on DNA topology is indirect, possibly due to the sRNA-based regulation of another protein affecting DNA supercoiling.

**Figure 5:**
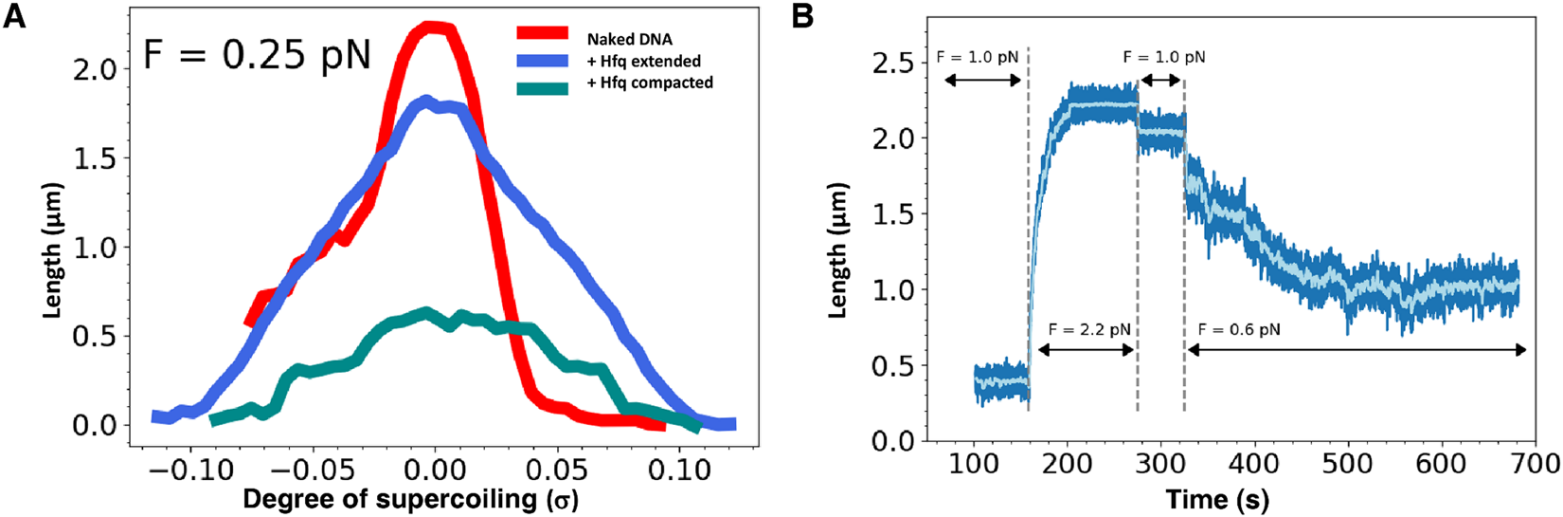
Real-time Hfq-DNA binding followed by magnetic tweezers. The absence of direct effect of Hfq has also been confirmed by tweezers experiments. Note that in this case we used a linear DNA. (**A**) Extension-versus-rotation curves at 0.25 pN of naked DNA (red), DNA associated with Hfq (present at a concentration of 60 nM) in extended (blue) and compact states (cyan). Compact conformation is accessible through application of supercoiling larger than +/−0.1, or after letting the molecule at low force (below 0.05 pN) for a few minutes. Asymmetry in the response of naked DNA is due to denaturation of the double helix under negative supercoiling. Hfq interactions prevent this denaturation. (**B**) Change of the length of DNA interacting with 60 nM of Hfq submitted to different stretching forces (light blue, running average over a 5 s window). At t = 100 s the molecule is compacted under a force of 1.0 +/− 0.1 pN and σ = 0. While the force is increased to 2.2 +/− 0.1 pN DNA undergoes a continuous lengthening until it reaches a plateau at 2.2 µm. Force was then further decreased, when set to 0.6 +/− 0.07 pN molecule relaxed towards a partially compact state with a length of 1.0 µm. As expected, truncated Hfq in the same experiment does not have any effect.

To confirm this result, we used magnetic tweezers to handle a single topologically constrained double stranded (ds) DNA molecule^46^ and monitor its deformation upon Hfq binding in real time. Rotation of the magnets imposed a variable amount of supercoiling to a single 8.8 kbase pair (bp) dsDNA molecule. The extension of the molecule is measured for multiple values of the supercoiling σ (defined as the number of turns added or removed relative to the total number of turns in the relaxed naked molecule), giving access to the extension-versus-rotation response of the molecule in the presence or absence of Hfq (Fig. 5)^46^. Maximal extension of naked dsDNA (2.25 µm) is reached when no supercoiling is applied (σ = 0) (Fig. 5A red line). The apex is flanked by extensive shortening regimes, due to formation of loops (plectonemes), either upon negative or positive supercoiling. Asymmetry in the response of naked DNA is due to denaturation of the double helix under negative supercoiling^46^. After this measurement, we injected a solution containing 60 nM Hfq without supercoiling (σ = 0). We then measured the extension-versus-rotation response of the molecule at a constant force of 0.25 pN (Fig. 5A, blue line) and observed a distinctive behaviour. The curve is still centered at σ = 0, indicating that no twist is applied to the DNA molecule through Hfq interaction. Hfq binding to DNA only slightly changes the maximal extension of the molecule under the same force (1.75 µm compared to 2.25 µm). This shortening could reflect a decrease in the persistence length of the filament^47^ or could be due to the formation of kinks in the dsDNA backbone by Hfq. The extension-versus-rotation curve profile of Hfq-DNA filament also displays an apex flanked by plectonemic regimes. Strikingly, the width of the apex is unchanged, indicating that the torsional stiffness is not affected by Hfq binding. Slopes of the plectonemic regime are smaller, consistent with a decrease in persistence length^48^. We then forced Hfq-DNA filament extension to be close to 0 µm by holding it for several minutes at low force and no supercoiling (F < 0.1 pN and σ = 0). Consequently the extension-versus-rotation curve (Fig. 5A, green) displayed a broad and flat profile still centered at σ = 0 with a maximal extension shortened to 0.6 µm. This reflects the ability of Hfq to bridge adjacent dsDNA segments, preventing full extension under a moderated force. This sequential analysis shows that Hfq binds to dsDNA independently and prior bridging.

Finally, in order to further understand Hfq binding mode, DNA compaction was also investigated through length-versus-time measurements (Fig. 5B). Starting from the compact state at σ = 0 (center of the green line in Fig. 5A), the force was increased from 0.1 pN to 2.2 pN. A continuous lengthening of the filament was observed up to a plateau at 2.2 μm. The force was then lowered to 1 pN, a force at which the extension of the molecule remained constant about 2 µm. The force was then further decreased to 0.6 pN, leading to the relaxation of the fiber towards a partially compact state with a length of 1 μm. Such a dynamic and reversible transition between compact and extended states can be interpreted as the breakage/formation of interactions between Hfq proteins linked to the dsDNA molecule. Hfq-Hfq binding is likely to be responsible for the stabilization of this compact state.

**Figure 6:**
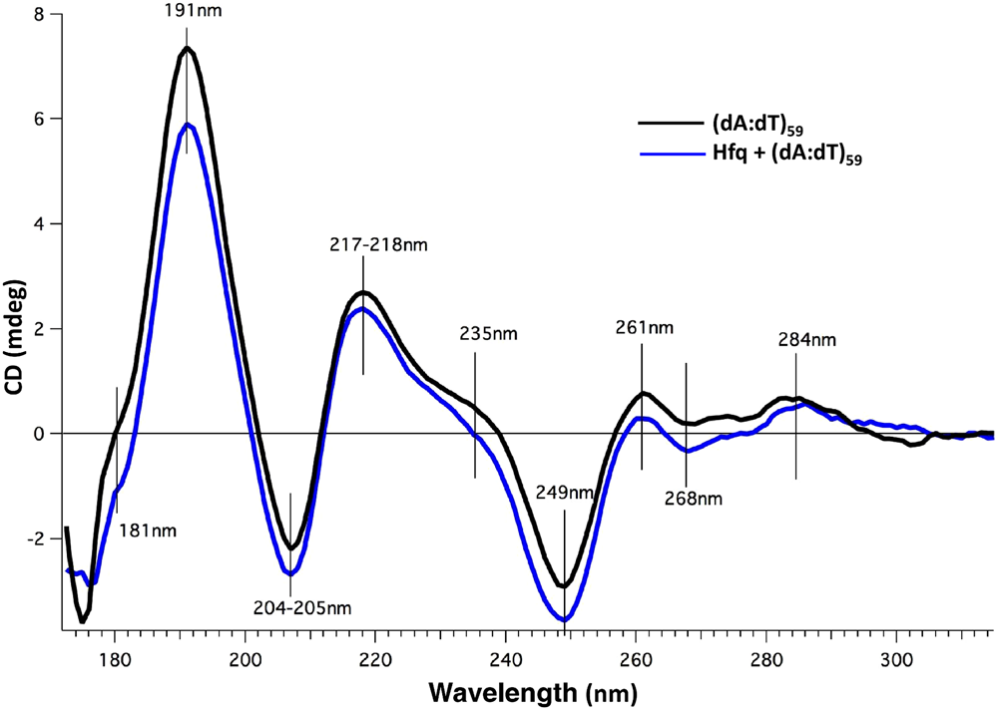
SRCD spectra of DNA interacting with full length Hfq. (dA:dT)_59_ with Hfq (blue line) or without Hfq (black line). The respective contribution of buffer and protein were subtracted. As shown in Fig. 1, spectral changes indicate that DNA induces a structural change of the protein and conversely. A low Hfq/bp ratio was chosen in order to minimize the signal of the protein sample and to focus on DNA structural change. As previously described^49^, the spectrum of the (dA:dT)_59_ duplex contains positive bands at ~217 nm and ~191 nm (with a shoulder at ~181 nm), and a negative band at ~205 nm. Spectral band differences for DNA with and without CTR at 181 and 191 nm (charge transfer and pi-pi*, respectively) correspond to local changes in DNA helix conformation. Further spectral differences include a shoulder at 235 nm and amplitude differences at 249, 261, 268 and 284 nm; the later three positions corresponding to well identified peaks in DNA CD spectroscopy reflecting the base pairing and stacking^49,84^. Smaller magnitudes reflect a weaker degree of base stacking^84^. Note that the amplitude differences are not proportional, which indicates that structural changes are occurring upon interaction between DNA and the CTR of Hfq.

As SRCD also allows to analyse DNA local conformation^49^, we investigated how full length Hfq affects (dA:dT)_59_ conformations (Fig. 6). A-tract was chosen as a model because Hfq preferentially interacts with deoxyadenosine rich sequences^12^. As expected, the spectrum of the (dA:dT) duplex contains positive bands at ~216 nm and ~190 nm, with a shoulder at ~180 nm, and a negative band at ~205 nm (Fig. 6). The unusual positive band at 190 nm is characteristic of A-tract conformation, which differs from the canonical B conformation^50^. In agreement with our previous report, the smaller bands at 180 and 190 nm in the presence of Hfq may represent base-pairing disruption and a local change in helix conformation^12^. Note that such a change in helix conformation may result in a change in DNA topology only if large regions of DNA are covered by Hfq, which is unlikely *in vivo*^13–15^ (especially because Dps is the major DNA binding protein during stationary phase^51^).

**Figure 7:**
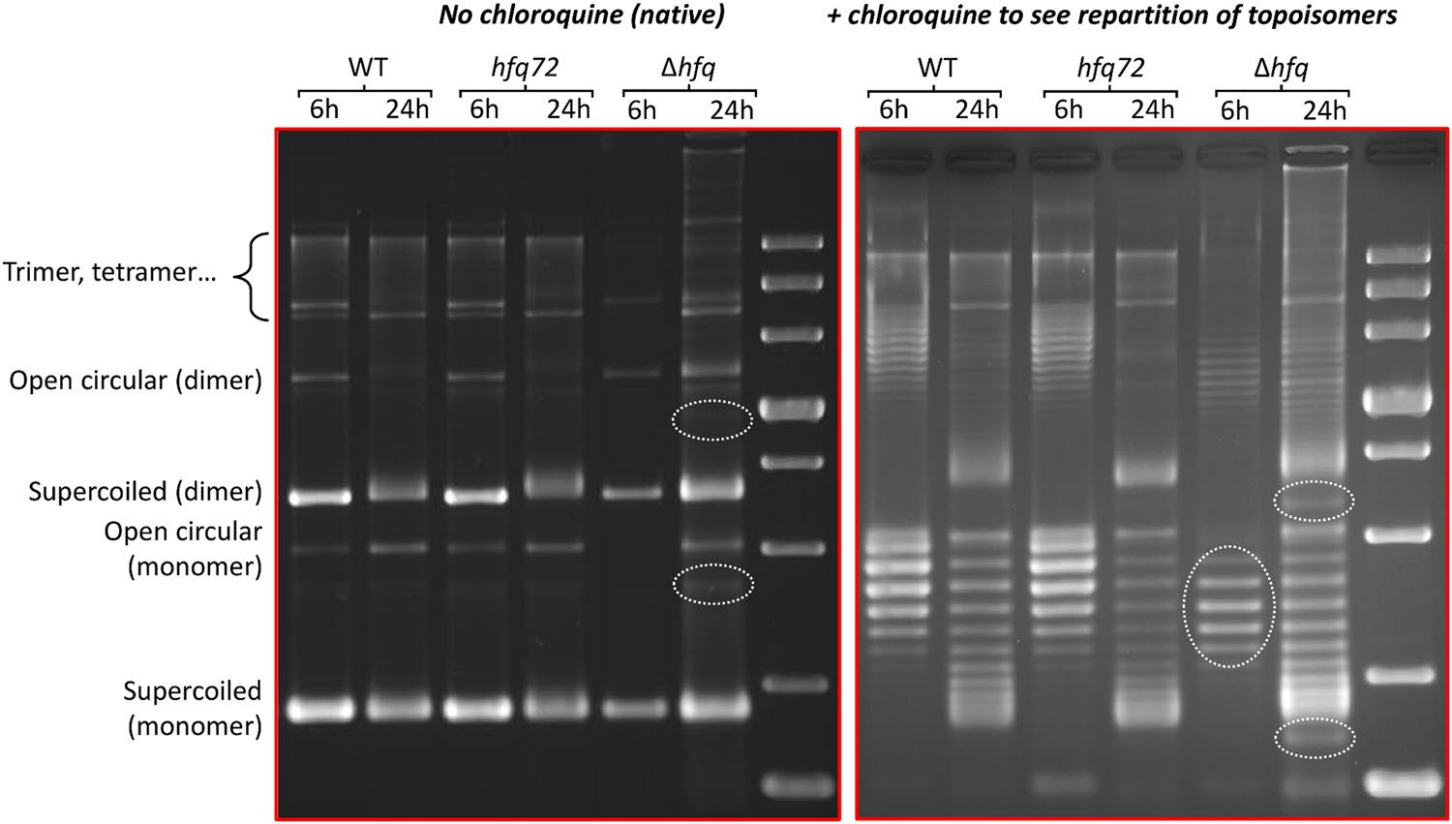
Topology gel of pHSG298 plasmids extracted from different strains. In the absence of chloroquine (native gel) relaxed plasmid goes slower, while in the presence of chloroquine (to see repartition of topoisomers) relaxed plasmid goes faster. Note that left and right parts are different gels stained separately. From this analysis we can conclude that plasmids are usually more relaxed at 24 h (stationary phase), that there is no difference between *hfqWT* and *hfq72*, indicating that strong compaction due to the CTR does not affect plasmid topology. Nevertheless, we also confirm that plasmids are more relaxed in Δ*hfq* strain than in others (see dashed ovals). The small bimodal population seen in Δ*hfq* strain at 24 h is comparable to the effect observed previously^2^. The presence of multimers is due to the use of MG1655 strain, which is *recA*+.

In order to go further in the mechanisms of Hfq-mediated DNA conformational change and the possible role of its CTR, we analysed the effect of Hfq on plasmid supercoiling *in vivo*. As Hfq is more abundant during the stationary phase, we focused our analysis on late exponential and stationary phases^15,37^. These phases were also those analysed in previous reports^2^. Note that it is difficult to compare WT and ∆*hfq* strains in the same region of exponential growth phase as both strains grow in a very different way. Topology gels of pHSG298 plasmids extracted from different strains (WT, *hfq72* = ∆CTR and ∆*hfq*) are shown on Fig. 7. Plasmid samples were electrophoresed on a native or chloroquine agarose gels to separate topoisomers. Under native electrophoresis conditions (Fig. 7 left), the more supercoiled they were, the quicker topoisomers migration was. In contrast to native gels, under the presence of chloroquine (Fig. 7 right), the most supercoiled topoisomers migrated slowly. From this analysis we can conclude that plasmids are usually more relaxed (=less negatively supercoiled) at 24 h (stationary phase) than at 6 h (late exponential phase). Nevertheless, we also observed that there is no difference between WT and *hfq72* strains, indicating that the strong compaction due to the CTR of Hfq does not affect plasmid topology. On the other hand, we confirm that plasmids are more relaxed in Δ*hfq* strain, as previously observed^2^.

### Hfq compaction influences plasmid replication efficiency

**Figure 8:**
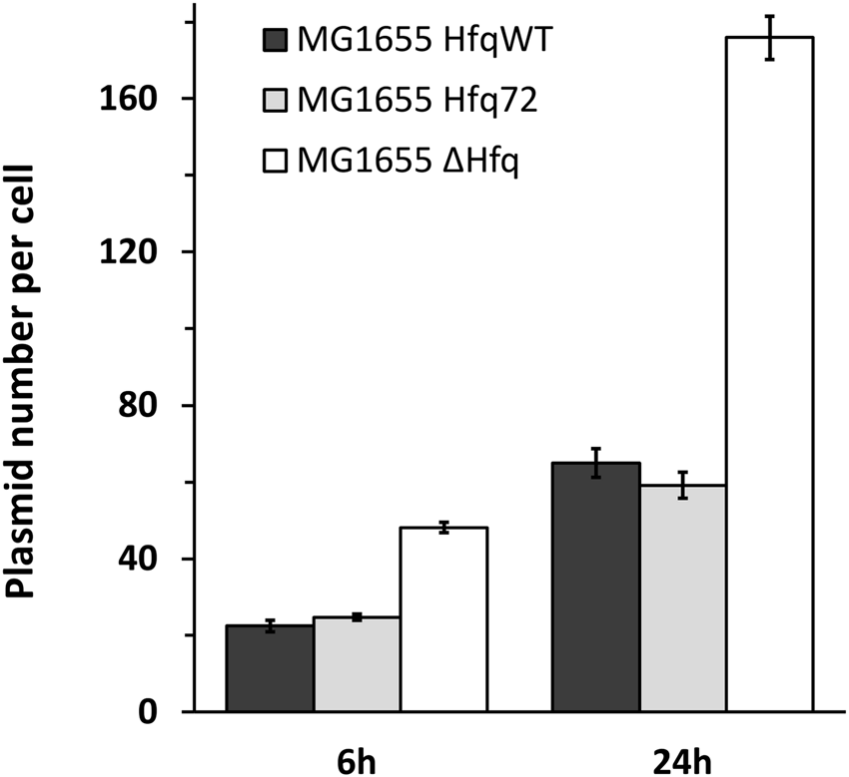
Evaluation of pHSG298 plasmid copy number in *hfq* mutated strains. As expected, a great number of plasmids is observed in late exponential and stationary phases and in the total absence of Hfq. This reconfirms the results of Cech *et al*.^37^ and shows additionally that deletion of Hfq CTR does not have an impact on plasmid copy number.

We then compared the effect on plasmid compaction and supercoiling to replication efficiency in the same phase of growth. As expected, our analysis confirms a greater number of plasmids in late exponential and stationary phases, and in the total absence of Hfq (Fig. 8)^37^. Under these conditions, *i.e*. total absence of Hfq, plasmids are not Hfq-compacted and hence are more relaxed. A possibility is that replication is more efficient due to these differences in DNA topology and compaction. In order to distinguish between these possibilities, we analysed the effect of Hfq-CTR deletion on replication efficiency. Total suppression of Hfq results in a difference for both supercoiling and compaction, while deletion of its CTR changes compaction only (no effect is observed on supercoiling, see above). As seen on Fig. 8, no significant difference is observed between WT and *hfq72* strains. Thus, our results indicate that the effect on replication efficiency is directly related to the change in DNA supercoiling, but independent of DNA compaction. If replication efficiency were dependent on compaction, deletion of Hfq-CTR in *hfq72* should also change replication efficiency.

## Discussion

In this manuscript we establish for the first time that Hfq self-assembly on DNA, responsible of DNA bridging and compaction, is due to the amyloid-like structure of its CTR. Furthermore we show that DNA induces amyloidogenesis of this region of the protein. In contrast to what has been observed in another extensively studied bacterial protein, RepA^52^, in the case of Hfq, DNA seems to be a constituent of the fibril. This conclusion is based on following observations: *(i)* peptide hydrogens are entrapped inside the complex with DNA and cannot exchange with deuterium (Fig. 2); *(ii)* DNA is found in the pellet upon centrifugation of fibrils prepared with DNA; *(iii)* this is confirmed with electrophoretic analysis where DNA is retained in the well in the presence of Hfq-CTR (see Malabirade *et al*.^24^), consistent with a stable fiber–DNA interaction; *(iv)* as previously observed, the CTR peptide surrounds DNA molecule with a succession of covered and naked DNA regions^13,24^, indicating the self-assembled CTR is bound to DNA; *(v)* increase of inter-sheet and inter-strand spacing in the presence of DNA (Fig. 3); *(vi)* DNA is partially protected and FTIR amyloid signal is reduced after DNAse I enzymatic digestion of the complex, strongly indicating that DNA is a component of the fiber. This contrasts with the case of RepA, where DNA was essentially in the soluble fraction after centrifugation, where the complex in native gels showed no retarded DNA in the well and where DNase I efficiently degraded most of DNA and does not alter the fibers^52^. Strikingly, RepA, has also been shown to bridge together two plasmid DNA molecules through an amyloid oligomeric structure, relevant to control plasmid copy number *in vivo*^53^. These two distinct proteins thus affect plasmid biology using a closely related mechanism (amyloidogenesis) but through direct or indirect pathways.

This new property opens perspectives about how Hfq, in addition to its role in RNA metabolism, binds DNA and may play a role in organising the bacterial chromosome by inducing a mechanical modification of the nucleic acid. Taking into account that a *E. coli* cell contains approximately 10 000 Hfq hexamers^54^, ~20% of which are found in the nucleoid^10,15^, the high amount of this protein gives a fairly good potential for DNA-bound domains. Hfq was previously shown to interact with DNA *via* its distal surface^11^, which is dedicated to A-rich sequence binding^20^; thus, one can speculate that the protein first binds and nucleates on A-containing sequences *via* its distal face^12^ (for instance in the A/T-rich region of some consensus sequences^11^) and then propagates to surrounding sequences. Consequently, Hfq may cover up quite large regions of DNA due to binding to the nucleic acid that generates a fiber-like pattern^12,24^. This is supported by the previous observation that Hfq self-assembles into filaments *in vitro*^31,33^ and presumably *in vivo*, as suggested by the fiber-like structures seen in the nucleoid by using TEM cellular imaging^13,15^.

**Figure 9:**
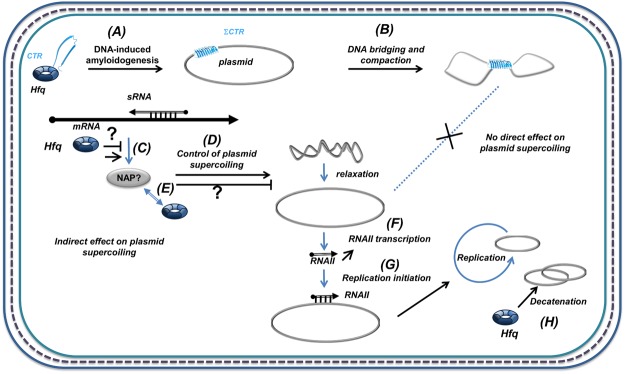
Hfq role in the control of plasmid compaction, supercoiling and replication. Hfq was previously shown to strongly influence DNA compaction (**B**)^23,24^. But Hfq also regulates at the post-transcriptionnal level the expression of various proteins, including NAPs such as H-NS or Dps^56,57^ (**C**). These NAPs can influence DNA topology^55^ (**D**). Here we demonstrate that DNA induces amyloidogenesis of Hfq-CTR (ΣCTR refers to amyloid self-assembly) (**A**), resulting in DNA bridging and compaction (**B**). In parallel, sRNA-dependent post-transcriptional regulation may regulate positively or negatively the expression of one NAP, resulting in changes in plasmid supercoiling (positive or negative). Note that Hfq and some NAP may interact physically or functionally (**E**). The global effect of Hfq deletion on topology results in plasmid relaxation, possibly due to primer (RNAII) transcription (**F**) involved in replication initiation (**G**) or in plasmids decatenation after replication (**H**). Hence, Hfq deletion influences plasmid replication positively. Plasmids are depicted as grey circles; non coding RNAs as open arrows; mRNAs as thick black lines; 5′ and 3′ of the mRNA are depicted by a “ball and arrow head”, respectively; Hfq as a toroidal hexamer; other proteins (NAPS) as grey ellipses; positive and negative regulation are indicated by arrows and horizontal bars, respectively; dotted line symbolizes Peptidoglycan (PG) between outer (OM) and inner (IM) membranes.

In this manuscript, we demonstrate for the first time that DNA itself promotes Hfq self-assembly into an amyloid-like structure. Self-assembly on DNA may thus have important consequences on the nucleic acid structure. One can expect effects on DNA compaction and supercoiling, and both have been reported earlier^2,23,24^. In this manuscript, we clarify the precise effect of Hfq on DNA compaction and topology. If compaction has been clearly assigned to direct Hfq DNA binding *in vitro*, and precisely to its CTR^23,24^, the effect of Hfq on supercoiling observed *in vivo* may be indirect. Indeed, Hfq regulates the expression of many proteins, including some affecting DNA topology^8,55^ (Fig. 9). Here we clearly establish that if Hfq compacts DNA thanks to its CTR amyloid region^23^, and if its CTR induces bridging of double stranded DNA distant sequences^12^, these processes do not result in a change in DNA supercoiling. Topological effects observed previously in ∆*hfq* strain are thus indirect: Hfq by itself does not change DNA twisting significantly and its impact on topology observed *in vivo* is likely due the riboregulation of another protein, such as H-NS or Dps^56,57^ (Fig. 9). Note that the bridging property reported for Hfq is parallel to H-NS self-assembly and bridging capacity^58^, the underlying mechanism of which was largely solved by single-molecule techniques^59–62^. H-NS uses a coiled-coil motif for its self-assembly^58^, while Hfq uses an amyloid-like structure. Hfq thus enlarges the bridging family of nucleoid-associated proteins^63^, even if in this case bridging results from the formation of an amyloid-like structure induced by DNA and does not result in an effect on overall DNA topology, in contrast with H-NS^45,55,64^. Note also that similarly to H-NS, the link between DNA-bridging and change in topology may be sequence-dependent^65^. Finally, both proteins have very similar sequence specificity^11,66^ and the way they could physically^67^ or functionally^56^ interact should be investigated further (Fig. 9).

Although the relation between DNA bridging, supercoiling and genetic regulation is still the subject of intense speculation^68,69^, our study provides new insights into the role of Hfq for replication efficiency^13,37^. The majority of plasmids replicate less efficiently in the relaxed form, as supercoiling is required to facilitate opening of the origin. However, in our case we used a ColE1-like plasmid^70^, for which replication depends on transcription and formation of the RNAI-RNAII complex^71,72^. We hypothesise that the effect observed for Hfq on replication efficiency is mainly due to its (indirect) role in DNA supercoiling and precisely, in its role in primer (RNAII) transcription (Fig. 9). Nevertheless, additional effects of Hfq on RNAI-RNAII annealing could also influence replication efficiency, besides its effect on primer transcription^73^. In addition, Hfq may also play a role in the segregation of plasmids, by reducing the linking number during elongation of DNA synthesis, similarly to DNA gyrase^74^.

In conclusion, although work remains to be done to decipher Hfq precise mechanism at stake, our results definitely demonstrate Hfq, a riboregulator, is an important player in bacterial chromosome structure, directly or *via* sRNA based regulations (Fig. 9). Furthermore, as pathologic amyloids have also gene-regulatory functions^75–77^, our work additionally opens new perspectives on the interaction of these amyloids with DNA in eukaryotic cells.

## Materials and Methods

### Expression and purification of Hfq proteins

Full length and truncated Hfq (residues 1–72) were expressed and prepared as described previously^24,36^. Hfq-CTR was chemically synthesised (Proteogenix, France)^33^.

### Preparation of the complexes for SRCD, FTIR and SAXS

Complexes between Hfq-CTR and (dA:dT)_59_ duplex (Eurogentech) were prepared in water and used at a final concentration of 1.8 mM and 7.3 mM, respectively. The stoichiometry was 1 Hfq-CTR per 4 base pair. For full length HfqWT and (dA:dT)_59_, 10 µM of Hfq were mixed with 2.23 mM of (dA:dT)_59_, giving a stoichiometry of 1 Hfq hexamer per 220 base pairs. A lower Hfq/bp ratio was chosen to minimize the signal of the protein sample in order to focus on DNA structural change. Samples were analysed at specific times. Note that when the complex is formed in the presence of salts and buffers, the spectral bandwidth accessible was limited, reducing the spectral information content. Nevertheless, comparison in the UV spectral range of complexes in water and salt did not show any spectral differences. Furthermore addition of Na^+^ at 50 and 250 mM did not allow to detect any effect on peptide self-assembly kinetics at the concentration used for our analysis, ruling out the possibility that the effect observed could be due to traces of salts. Besides, addition of salt may result in complex disassembly^78^.

### Synchrotron Radiation Circular Dichroism (SRCD)

For SRCD analysis, measurements and data collection were carried out on DISCO beam-line at the SOLEIL Synchrotron (proposal 20171061)^79^. After different incubation time, 2–4 µl of samples were loaded into circular demountable CaF_2_ cells of 33 microns path length^80^. Three separated data collections with fresh sample preparations were carried out to ensure consistency and repeatability. Spectral acquisitions of 1 nm steps at 1.2 integration time, between 320 and 170 nm were performed in triplicate for the samples as well as for the baselines. (+)-camphor-10-sulfonic acid (CSA) was used to calibrate amplitudes and wavelength positions of the SRCD experiment. Data-analyses including averaging, baseline subtraction, smoothing, scaling and standardization were carried out with CDtool.

### Fourier Transform Infrared spectroscopy (FTIR)

For FTIR analysis, the same solutions used for SRCD analysis were lyophilized and re-dissolved in D_2_O (5 µL). FTIR spectra were recorded using a Bruker Tensor 27 spectrophotometer. For transmission experiments, samples were deposited between two ZnSe windows without spacer. 30 scans were usually accumulated under continuous dry air purge, with a resolution of 1 cm^−1^. Data treatment was performed using the OPUS software (Bruker) and consisted of multiple point base line correction and spectral subtraction after normalization.

### Small Angle X-ray Scattering (SAXS)

SAXS measurements were performed on the high brilliance SWING beam line at the Soleil synchrotron facility, with monochromator set at 12 KeV (proposal 20170388) using an EIGERX-4M detector at 0.560 m from the sample^81^; diffraction patterns were recorded for reciprocal spacing q = 4 π sin(θ)/λ varying between 0.02 and 1.72 Å^−1^, that is, repetitive distances d = 2 π/q ranging from 3.14 to 3.65 Å. SAXS pattern of samples and references were recorded in previously filled Ø1.5 mm quartz capillaries. 10 patterns were acquired successively for each sample, thus exposure time was tuned in order to avoid radiation damage. 1D SAXS curves were obtained by circular averaging of the 2D images using Foxtrot software (http://www.synchrotron-soleil.fr/Recherche/LignesLumiere/SWING). As no difference was observed between the 10 images recorded for each samples, the 1D curves were averaged in order to obtain 1 curve for each samples and references with better statistics.

### Construction of *E. coli* strains

Strains were constructed with the λ-red recombination technique^82^. Briefly, the *E. coli* MG1655 strain was transformed and grown with the temperature sensitive pKD46 plasmid containing Red recombinase expression genes, in presence of arabinose. PCR fragments containing the desired mutation plus an antibiotic resistance cassette were electropored to allow recombination. Bacteria were selected for antibiotic resistance and the introduction of the mutation was checked by PCR and sequencing.

First, a reference strain (MG1655 *hfq*:*cat*) was obtained by inserting a chloramphenicol resistance cassette downstream the *hfq* ORF of MG1655, using the pKD3 plasmid as PCR template. Primers sequences were 5′-AATACTTCCGCGCAACAGGACAGCGAAGAAACCGAATAAGGTTCCATGGTCCATATGAATATC-3′ (forward) and 5′-AGGATCGCTGGCTCCCCGTGTAAAAAAACAGCCCGAAACCTGTGTAGGCTGGAGCTGCTT-3′ (reverse). Then, a second strain deleted for the last 90 *hfq* nucleotides giving a truncated protein with only the first 72 amino-acids (MG1655 *hfq72*:*cat*) was obtained by the same technique but using the previous strain as a template for amplifying the DNA fragment. Recombination was performed in the MG1655 strain. Primers used were 5′-ATTTCTACTGTTGTCCCGTCTCGCCCGGTTTCTC ATCACAGTTAAGGTTCCATGGTCCATATGAATATC-3′(forward) and 5′CGCTGGCTCCCCGTGTAAAAAAACAGCCCGAAACC-3′ (reverse).

### *In vitro* analysis of plasmid topology

The experiments were performed as described in Tupper *et al*.^45^. 5 µg of purified pHSG298 (2675 bp, Kan^R^, Takara Bio) plasmid was incubated 10 min at room temperature with full length Hfq in a total volume of 100 µL of buffer containing 10 mM Tris-HCl pH 7.5, 0.1 mM EDTA, 15 mM KCl, 2 mM spermidine, 15% v/v glycerol and 0.1 g.L^−1^ BSA. 18U of calf thymus topoisomerase I (Invitrogen) were then added and the incubation continued for 30 min at 37 °C. Proteins were extracted with ultrapure phenol/chloroform/isoamyl alcohol (25:24:1 v/v, Invitrogen) and ethanol precipitation. The DNA was re-suspended in 50 µL Tris-HCl (5 mM, pH8). 2 µL of purified plasmids were electrophoresed in a 1% w/v native agarose gel. Electrophoresis was performed in Tris-Borate-EDTA 1X, 16.5 V/cm during 30 min. The gel was then rinsed 15 min with water before staining 30 min with GelRed fluorescent dye (Biotium, Inc.). Images were acquired with a G-box (Syngene) imager.

### ***In vivo*** analysis of plasmid topology

Strains transformed with pHSG298 were grown overnight in LB with kanamycin at 37 °C with good aeration and shaking. The next day, precultures were refreshed by 100X dilution in 50 mL cultures and grown in 250 mL Erlenmeyers flasks at 120 rpm. At 6 h and 24 h of cultivation time, pHSG298 was purified from 5 mL culture samples with NucleoSpin Plasmid EasyPure extraction kit (Macherey Nagel), and eluted in Tris-HCl (5 mM, pH8) buffer. Each plasmid purification method may have potential biases concerning DNA topology selection, but we preferred to use a kit based on silica column to compare our results with those of Tsui *et al*.^2^. In addition, all samples were processed the same way and the only difference was the genetic background. Around 0.3 µg of purified plasmids were electrophoresed in 1% w/v agarose gels in the presence or the absence of 10 µg.mL^−1^ chloroquine to separate the plasmids of different linking numbers (Lk). Electrophoresis was performed in Tris-Borate-EDTA 1X, 2.5 V/cm during 16 h at 8 °C. The gel was then incubated 1 h in 10 mM MgSO_4_ to remove excess of chloroquine (when appropriate), and otherwise stained and observed as above.

### Magnetic tweezers experiments

A poly-di-methylsiloxane (PDMS; Dow-Corning) flow cell with a 2-mm-wide and 100-µm-high channel was mounted on a glass coverslip previously silanized with Sigmacote (Sigma). Anti-digoxigenin (Roche) was flushed into the channel and bound non-specifically for 1 h at 37 °C, followed by overnight blocking with BSA. The PDMS flow cell was placed beneath two permanent NdFeB magnets. Image acquisition utilized a 60 Hz camera (A10 GigE; JAI) placed on an inverted microscope (Picotwist). Image analysis was performed with Pico software. The 8.8 kb DNA molecule held in magnetic tweezers setup was prepared by PCR from template lambda-DNA, it was then ligated at each end with a 600-bp DNA fragments prepared by PCR from template Litmus28i (NEB; positions 2008 and 2580), one was modified with biotin-tagged dUTP and the other with digoxigenin-tagged dUTP (Roche Diagnostics). This DNA fragment was subsequently attached to a magnetic bead coated with streptavidin prior injection of this construct into the microchannel and attachment to the antidigoxigenin-coated surface. Experiments were carried in buffer A (10 mM Tris-HCl pH 7, 1 mM EDTA, 0.1 mg/mL BSA). Hfq concentration was chosen as follow: we know that a ratio ~50 monomeric Hfq/DNA molecule allows having a significant coverage of DNA molecule^12,13^. Similarly this assay is done with highly diluted, single DNA molecules to provide information about intramolecular compaction. We previously found that compaction requires an order of magnitude lower of the CTR fragment than the one pertaining to 50% binding saturation^24^. For this reason, we used 60 nM of Hfq for our analysis, corresponding to ~50 Hfq in the volume explored around a 2 µm-long-DNA attached to the glass coverslip.

### qPCR plasmid quantification

The different strains transformed with pHSG298 were grown overnight in LB supplemented with kanamycin at 37 °C with good aeration and shaking. The next day, precultures were refreshed by 100X dilution in 50 mL cultures and grown in 250 mL Erlenmeyer flaks at 120 RPM. At 6 h and 24 h of cultivation time, 3 mL samples for each strain were centrifuged at 5000 g. The pellet was rinsed twice with PBS and re-suspended in 0.5 mL PBS 1X. Samples were lysed at 95 °C during 5 min, and centrifuged again to remove cellular debris before being subjected to qPCR.

For the quantifications, samples were diluted and mixed with Brilliant III Ultra-Fast SYBR Green qPCR Master Mix (Agilent Technologies) and 300 µM primers according to the manufacturer recommendations. Oligonucleotides targeting regions in *mreB* gene (chromosome) and *kan*^*r*^ (plasmid) DNA sequences, plasmids harbouring target sequences used at known copy numbers for calibration curves and qPCR protocol are described elsewhere^9^. Quantities inferred from Cp were determined and averaged from 8 replicates for the *kan*^*r*^ gene and were divided by the quantities obtained for *mreB*, giving the number of pHSG298 copies per cell.
